# Giant Ectopic Ureter Mimicking Pelvic Organ Prolapse: A Case Report

**DOI:** 10.1155/2011/304917

**Published:** 2011-09-11

**Authors:** Adnan Simsir, Fuat Kizilay, Bilbasar Yildiz, Oktay Nazli

**Affiliations:** Department of Urology, Ege University School of Medicine, 35100 Izmir, Turkey

## Abstract

Ectopic ureter is one of the most common urinary tract anomalies. We, herein, present a case of a giant ureter with ectopic orifice, mimicking pelvic organ prolapse, which is the first in the literature. A 59-year-old female patient presenting with frequently recurrent urinary tract infection had grade 3 pelvic organ prolapse. On examination, the organ producing the appearance of prolapse was found to be a right ureter of giant size and was obstructed by a large stone at the distal segment. The proximal end of the ureter ended blindly. After exploration, the stone was removed, the ureter was detached from the urethra, and the lumen was tied off and cut 5 cm proximally. At 6 months postoperatively, the patient is being followed up without any clinical problems. In such cases with nonfunctioning renal segment draining proximally, the chance of cure can be obtained without a need for a comprehensive intervention such as total abdominal ureterectomy.

## 1. Introduction

Complete or incomplete ureteral duplication is the most common congenital malformation of the urinary tract, with an incidence reported to be 0.9% in autopsy series and 2–4% in clinical series [1]. Ureteral duplication may be complete or incomplete, whereas there may be no clinical findings in most cases of incomplete ureteral duplication. In cases of complete ureteral duplication, there are two totally separate ureters and two separate renal pelves. These cases usually present with frequently recurrent infections [2]. In this condition, which is more common in females, the ureter draining to the upper pole is usually ectopic and may open distal to the external sphincter, even outside the urinary tract [3].

## 2. Case Report

A 59-year-old female patient was admitted to our hospital with complaints of recurrent right side pain, voiding difficulty, and frequently recurrent urinary tract infection. Physical examination revealed grade 3 pelvic organ prolapse (POP) as well as anterior wall defect. Additionally, on vaginal examination, there was a mobile, firm, palpable mass of approximately 15 mm in diameter. Urodynamic investigations revealed stress incontinence and no postvoiding residual urine. Medical history of the patient included continuous urinary incontinence in childhood, which, however, was reported to have been disappeared in subsequent years.

**Figure 1:**
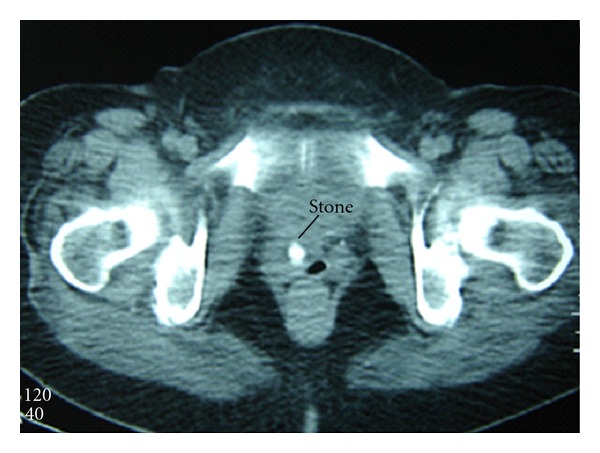
Urethral stone in MRI.

Magnetic Resonance (MR) urography showed irregularity of the upper pole of the right kidney and a diverticulum-like formation with a 12 mm stone inside (Figure 1).

**Figure 2:**
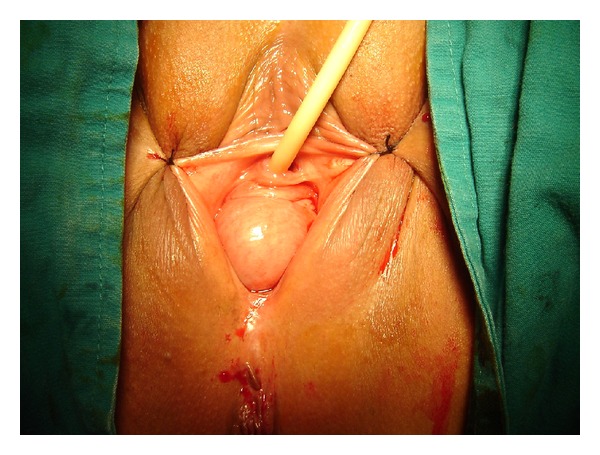
Ectopic ureter, mimicking POP.

In the light of these data, the patient was planned to undergo removal of the stone inside the diverticulum as well as excision of the diverticulum and cystocele repair. With the patient under spinal anesthesia, a 15 Fr flexible cystoscope was inserted into the urethra, and the diverticular orifice was visualized at the 7 o'clock position proximal to the urethra (Figure 2).

**Figure 3:**
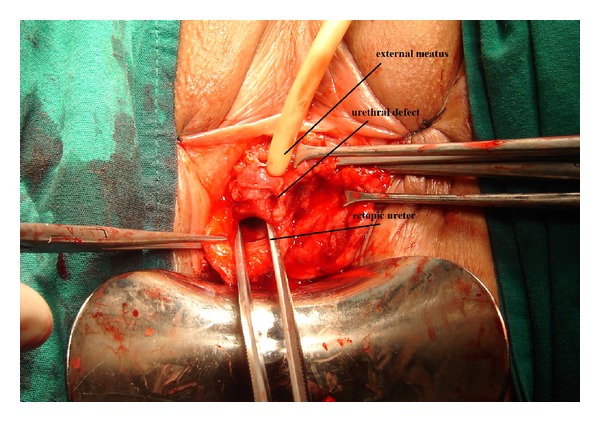
View after exploration.

 The stone in the diverticulum was visualized and removed using a forceps. Subsequently, seeing that the diverticulum extended proximally, the decision to perform ureteroscopy was made. The ureteroscope was advanced 20 cm from this pouch of 6 cm in diameter which was found to end blindly. Here, radiopaque material was injected, and a radiograph was taken which showed a giant ectopic ureter of 20 cm length, ending blindly. Subsequently, the bladder was entered. The ipsilateral ureteral orifice was identified, radiopaque material was injected here, and a radiograph was taken which revealed no apparent abnormality. Considering that we are faced with a considerably dilated ureter with ectopic opening, draining the upper pole of the kidney, conversion to open surgery was decided. With the patient placed in the lithotomy position, transvaginal incision was performed. The dilated ureter was identified just beneath the vaginal wall and was freed. The dissection was advanced distally through the urethra. It was separated from the urethra with sharp dissection. The urethral defect was closed using absorbable sutures. The ureter was freed as much as possible using a transvaginal approach. In the meanwhile, it was observed that the patient had no POP, and the mass in the vagina was induced by the giant ureter. The ureter was tied off and cut 5 cm proximally (Figure 3). Subsequently, the vaginal wall was closed using double-layer absorbable suture line. At 6 months postoperatively, the patient is still being followed up without any complications. In the postoperative period, the complaints of vaginal mass and stress incontinence were resolved completely.

## 3. Discussion

An ectopic ureter is defined as an ureter which opens anywhere other than the normal position. It is more common among females and is usually associated with double collecting system [4]. The location of the ureteral orifice is important with regard to the presentation of symptoms. The classic presentation of an infrasphincteric orifice is continuous incontinence with normal voiding pattern. In suprasphincteric orifice, the diagnosis is usually established during investigation of symptoms due to obstruction or recurrent infections [5]. In our case, the patient's complaint of incontinence in the past and, later, the detection of irregularity of the ipsilateral renal upper pole suggest the presence of an infrasphincteric lesion which is considered to have been disappeared as a result of function loss of the upper pole in years. Because the segments drained by ectopic ureters are usually dysplastic or hypoplastic, heminephrectomy is preferred [6], whereas there was no need for such an intervention in our case since the ectopic ureter was not attached to the renal system and ended blindly, and the ureter ending blindly was excised via a transvaginal approach.

As far as we are aware, this is the first case of ectopic ureteral opening mimicking pelvic organ prolapse in the current literature. When compared to similar cases, it should be taken into consideration that the patient can be treated without need for a thorough and comprehensive surgery by tying off the distal portion of the ureter in the case of an ectopic ureter arising from a nonfunctioning renal segment.
